# Aberrations in SMAD family of genes among HNSCC patients

**DOI:** 10.6026/973206300171113

**Published:** 2021-12-31

**Authors:** E Thariny, AM Smiline Girija, A Paramasivam, J Vijayashree Priyadharsini

**Affiliations:** 1 Saveetha Dental College, Saveetha Institute of Medical and Technical Sciences (SIMATS), Saveetha University, Chennai, India

**Keywords:** Cancer, mutation, SMAD gene, genetic alteration, head & neck, pathogenicity

## Abstract

Head and neck cancer is a debilitating disease with several etiological factors. One of the main etiologies to be noticed is the alteration, which is either caused by genetic or environmental factors. Therefore, it is of interest to assess the effect of genetic alterations, especially the non-synonymous mutations of the SMAD gene family and its possible association with HNSCC. Data shows a significant novel mutation in the SMAD gene family in association with head and neck squamous cell carcinoma (HNSCC), which would aid in better diagnosis and treatment planning for cancer.

## Background:

Head and neck cancer is a morbid lethal malignancy. It is a cancer-causing growth that is present in the mouth, nose, throat, larynx, sinuses, or salivary glands [1]. Squamous cell carcinomas are more prevalent in human cancer with few therapeutic options such as chemotherapy and radiation. It is the second most common form of cancer in India among males [2]. Head and neck cancer is noted for more than 650000 cases and 330000 deaths annually which are recorded all over the world. In the United States, about 3% of cancers are head and neck cancer [3]. Overall the occurrence of head and neck cancer accounts mainly in oral cancer, which is 11.4 per 100000 persons per year worldwide. Members of the SMAD family include eight different SMAD in mammalian cell populations, which are classified into three categories: the first category includes the Receptor-regulated or regulatory SMAD gene (R-SMAD), which includes Smaad1, SMAD2, SMAD3, SMAD5, SMAD6, and SMAD7. The second category includes common SMAD (Co-SMAD), i.e SMAD 4.Third category includes inhibitory or anti-SMAD (I-SMAD), which areSMAD6 and SMAD7 [4]. The function of the SMAD gene is to deliver extracellular signals from TGF-beta ligands to the nucleus leading the activation of downstream gene transcription to regulate cell growth and division process. Ligand-induced activation of TGF- β family receptors with intrinsic serine/threonine kinase activity triggers the phosphorylation of receptor-regulated SMADs. The SMAD genes are expressed according to the categories, regulatory SMAD and common SMAD are located in the cytoplasm, but it gets accumulated once the TGF- β signaling is passed, where they can bind to DNA and regulate transcription. Inhibitory SMAD is predominantly found in the nucleus, where they can act as a direct transcriptional regulator [5]. The main function of the SMAD gene is to regulate the growth progression any change in the signaling pathway can mislead the outcome of the function. Alterations in the SMAD4 gene were found to be most often associated with head and neck cancer among the SMAD gene family [6]. Therefore, it is of interest to assess the effect of genetic alterations, especially the non-synonymous mutations of the SMAD gene family and its possible association with HNSCC.

## Materials & Methods:

### Data source:

The source of the patient's data was obtained from the cBioportal database [7,8]. This database contains an exhaustive collection of HNSCC case details from different cohorts. The TCGA, Firehose legacy data set constituted a total of 528 head and neck squamous cell carcinoma cases in which sequencing and copy number alteration data were presented for 512 tumor samples. A complete profile of mutated, amplified, deleted genes was in stock for each and every case in the dataset. The demographic details of the cases have been provided in (Table 1 - see PDF). A complete list of essential genes related to the SMAD gene family was derived from the "HUGO Gene Nomenclature Committee at the European Bioinformatics Institute" (www.genenames.org/data/) database. User-defined queries based on these genes were submitted to the cBioportal database and the resultant Oncoprint data was used for further analysis.

### Oncoprint data analysis:

**Figure 1:**
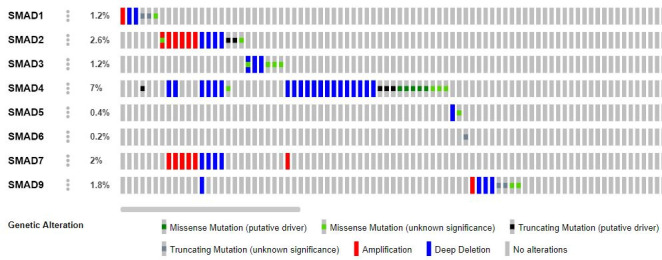
The oncoprint data depicting different types of genetic alteration in the SMAD family of genes. The SMAD4 was found to have the highest alteration level of all genes examined (7%). SMAD2 and SMAD7 genes were found to have numerous sites of amplification.

The Oncoprint data provides information on the frequency distribution of variations in each of the genes selected, type of variation, changes in the protein-coding amino acids, gene amplification, deletions, insertions, frameshifts, splice site mutations, etc. These details can be used to detect (a) derive a putative association between the disease phenotype and genotype, (b) identify the variations in less understood pathways or genes, and (c) identify any novel variations which can be associated with the disease phenotype (Table 2 - see PDF) (Figure 1).

### Protein stability analysis:

I-Mutant v3.0 is a support vector machine (SVM)-based tool for the automatic prediction of protein stability changes upon single point mutations. The software's predictions are based on the protein sequence. The free energy change (DDG) predicted by I-Mutant 3.0 is based on the difference between unfolding Gibbs free energy change of mutant and native protein (kcal/mol) [9] (Table 3 - see PDF).

### PROVEAN analysis:

Protein variation effect analyzer was used to predict whether the single nucleotide substitutions (non-synonymous variants) in the protein affect the protein function [10,11](Table 3 - see PDF).

### gnomAD analysis: 

gnomAD v2.1.1 dataset consists of a collection of 125,748 exomes and 15,708 genomes from human sequencing studies. This data was used to verify whether the missense variants found in the HNSCC data is prevalent in other individuals for whom the sequencing of gene data is available [12].

### UALCAN analysis:

UALCAN is a comprehensive, user-friendly, and interactive web resource for analyzing cancer OMICS data. The gene expression profile of genes of the SMAD family and the survival curve analysis for the same was performed with the TCGA dataset in UALCAN database [13].

## Results and Discussion:

### Oncoprint analysis:

Oncoprint analysis revealed the presence of deep deletions, amplifications, truncated, synonymous and non-synonymous variants. A few of them were found to be putative drivers. SMAD4 was found to harbor the highest frequency of alterations among all the genes analyzed (7%). Most of the alterations in SMAD4 were of deep deletion and missense type (Figure 1). Further, the variants observed in the present study were compared to the non-synonymous variants in the gnomAD database to identify whether the variant is novel or reported in the general population. The comparative analysis identified a few reported SNPs such as rs762012589in SMAD3 and rs553369182 in SMAD9 genes. In addition, several putative drivers precipitated by loss of function of genes such as Q455*, R182* of SMAD2 gene and Q248*, S242*, Q461* and Q450* of SMAD4 gene were also identified. Apart from these alterations R361H has been predicted to be oncogenic with a significant loss of function, while, A118V, W99C, Q366k, and R97C were statistically significant hotspots predicted to be oncogenic (Table 2 - see PDF).

### Protein and pathogenicity analysis:

The stability of proteins harboring variants as assessed by I-Mutant showed decrease in stability upon substitution with the nonsynonymous variant. PROVEAN analysis predicted M327I of SMAD2, Q366K and P298S of SMAD4 and P185S of SMAD9 to be neutral, whilst all other variants were found to be deleterious. W99C of SMAD4 gene was observed to show the lowest free energy value of -1.67 and lowest score of -11.75 depicting highly deleterious consequences among all the other variants (Table 3 - see PDF).

### Gene expression and survival curve analysis:

**Figure 2:**
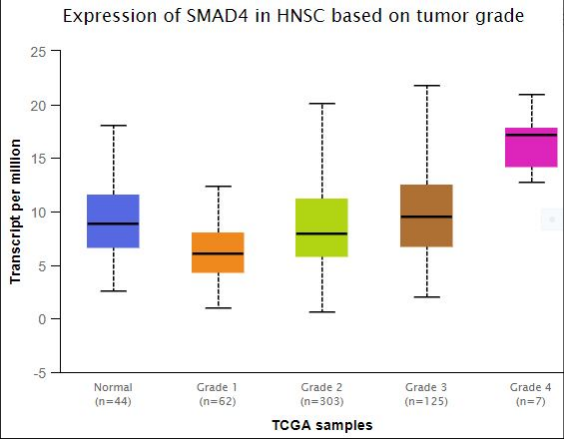
Box-Whisker plot representing the differential gene expression pattern of the SMAD4 gene across different tumor grades. The X-axis represents the different grades of HNSCC example samples from the TCGA data set and the Y axis denotes the transcripts per million values. A significant difference in the gene expression profile was observed between normal vs grade 1 (p = 1.49 x 10-4 ), normal vs grade 3 (p = 9.5 x 10-12), normal vs grade 4 (1.11 x 10-6), grade 1 vs grade 2 (p = 2.166 x 10-4 ), grade 1 vs grade 3 (p = 2.90 x 10-7), grade 1 vs grade 4 (p = 4.39 x 10-8), grade 2 vs grade 3 (p = 7.44 x 10-3), grade 2 vs grade 4 (p = 5.77 x 10-4) and grade 3 vs grade 4 (p = 1.98 x 10-2). A p-value of less than 0.05 is considered to be significant.

**Figure 3:**
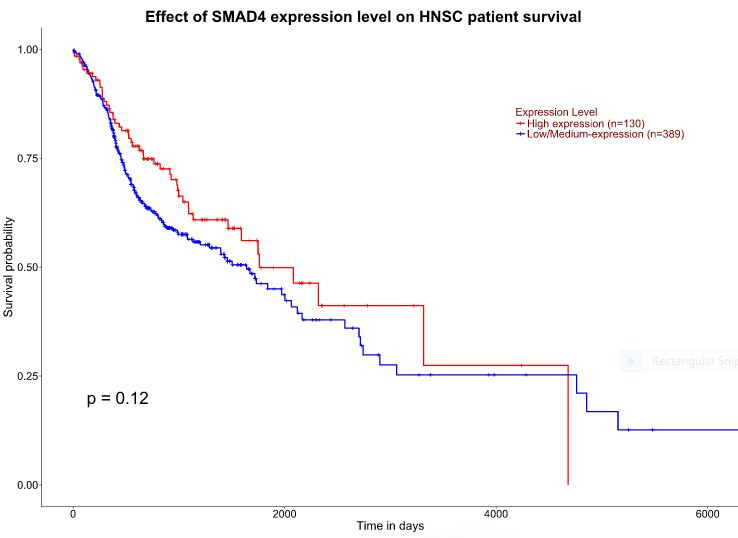
Kalplan-Meier plot showing the association of altered SMAD expression with HNSCC patients' survival. The X-axis represents time in days and Y-axis represents survival probability in HNSCC patients. The red line corresponds to high-level expression and the blue line corresponds to low/ medium level expression of SMAD (0.12). A p-value of more than 0.05 is not considered to be significant.

Since SMAD4 gene harbored the highest frequency of gene alteration especially deep deletions, further gene expression analysis was warranted to assess the effect of deletions. Differential expression of SMAD4 was found in different grades of the tumor as assessed using the TCGA data set in the UALCAN platform (Figure 2). Subsequent survival curves analysis although insignificant (p-value = 0.12) revealed that a higher level of SMAD4 expression provided a better survival advantage to the HNSCC patients when compared to those with low-level expression (Figure 3).

Varying rates of SMAD gene mutation has been detected worldwide in relation to head and neck cancer [14]. Defect in SMADsignaling can result in TGF-β resistance, leading to dysregulation of cell growth. This dysregulation stems into different forms cancers including pancreatic, colon, breast, and lung, oral and prostate cancer. SMAD4was first termed deleted in pancreatic cancer locus 4 (DPC4) [15-17]. Transforming growth factor β (TGF-B) is a vital component regulating the epithelial cell proliferation, cell division, immune function, and angiogenesis. Since, TGF-β signaling maintains epithelial homeostasis, any dysfunction in TGF-β signaling pathway can lead to malignancies [18]. Any abnormal alteration in SMAD gene causes defect in TGF-beta pathway, which results in hyper proliferation, reduced apoptosis, and increased genomic instability [19]. In order to compensate this, there is an abnormal increase in TGF-beta production by tumor epithelial cells, which further promotes tumor growth, and metastasis by increasing angiogenesis and inflammation in tumor stromal cells. It is noted that overexpression of SMAD7 causes oral epithelial dysplasia [20].

The loss of SMAD4 gene protein causes a high degree of instability in tumor epithelium. So, alteration in the SMAD gene leads to HNSCC tumorigenesis by blocking the growth prognosis and programmed cell death (apoptosis) which is usually controlled by TGF-β signaling pathway [21]. The oncoprint data analysis revealed deep deletions observed in the SMAD4 gene. It is noted that SMAD gene also has a role in epithelial-mesenchymal transition in which TGF-β functions as a transcriptional repressor of E-Cadherins, which are activated by the SMAD4 gene. Germline mutation of SMAD4 causes juvenile polyposis syndrome [22]. Recent studies have found that frequent deletion at loci 18q where the SMAD4 gene protein is the present and heterozygous loss of SMAD4 causes cancer [23]. It is found that nearly 61.12% of oral squamous cell carcinoma is related to the loss of SMAD4 protein [24]. SMAD1 alteration includes melorheostosis and osteopoikilosis [25]. This disease condition is associated with protein metabolism and the Th1 differentiation pathway [26]. It was analyzed that SMAD2 over expression of tumor-derived missense mutation was found to promote TGF-beta mediated invasion of MDCK (Madin-Darby canine kidney) cells [27]. The MH1 domain was frequently mutated in SMAD2 and SMAD4 alteration in pancreatic cancer. SMAD 7 expression was evident in oral dysplasia and also this specific gene acts a key negative regulator of the TGF-beta signaling pathway [28]. Interestingly, it has been demonstrated that one of the red complex pathogen Porphyromonas gingivalis is capable of promoting the progression of esophageal squamous cell cancer through TGF-betadependent Smad/YAP/TAZ signaling [29]. Limitations such as (a) the population or the data set is representative of a predominant group of individuals from a specific location which might not represent the cases observed throughout the world, (b) the habits of individuals differ in different geographical locations which may affect signaling pathways other than SMAD to precipitate the disease, (c) the identified variants have to be screened in other populations so as to arrive at conclusive evidence on the role of SMAD proteins in the pathogenesis of HNSCC should be noted.
